# Large bowel perforation secondary to endometriosis

**DOI:** 10.1093/jscr/rjad455

**Published:** 2023-08-14

**Authors:** Lawrence Y Goriel, David J Seok, Silvy C Akrawe, Yousif H Goriel

**Affiliations:** Department of General Surgery, Ascension Providence Hospital and Medical Center, Southfield, MI, USA; Department of General Surgery, Ascension Providence Hospital and Medical Center, Southfield, MI, USA; Department of General Surgery, Ascension Providence Hospital and Medical Center, Southfield, MI, USA; Faculty of Department of Surgery, Ascension Providence Hospital and Medical Center, Southfield, MI, USA

## Abstract

Endometriosis is described as the implantation of ectopic, viable endometrium. Among the complications associated with this phenomenon, ectopic foci that localizes to the bowel can result in many presentations. An uncommon presentation of such an occurrence is a mass effect on the colon causing an obstruction. This case report describes the progression of endometriosiscausing mass effect in the colon and resulting in perforated hollow viscous. Hence, this demonstrates the importance of maintaining endometriosis as a differential diagnosis in women of childbearing age presenting with bowel obstruction. Although the presence of endometriosis as a cause of bowel obstruction has been reported in the literature, the presentation of large bowel perforation is rare. In this case, an extremely rare presentation of sigmoid obstruction with transverse colonic perforation is observed.

## INTRODUCTION

Endometriosis is a common medical issue affecting up to 10% of females [1]. The most common presentation is the prodrome of dysmenorrhea followed by other complications related to the site of implantation of the ectopic tissue, which can include dyspareunia, dysuria and chronic abdominal pain [2]. Our patient, who had a known history of chronic constipation, presented to the emergency department in septic shock with severe abdominal distention and peritonitis. The purpose of this case report is to elucidate the importance of maintaining high suspicion, and perhaps more aggressive management, of endometriosis found to be implanted onto the intestines.

## CASE REPORT

**Figure 1:**
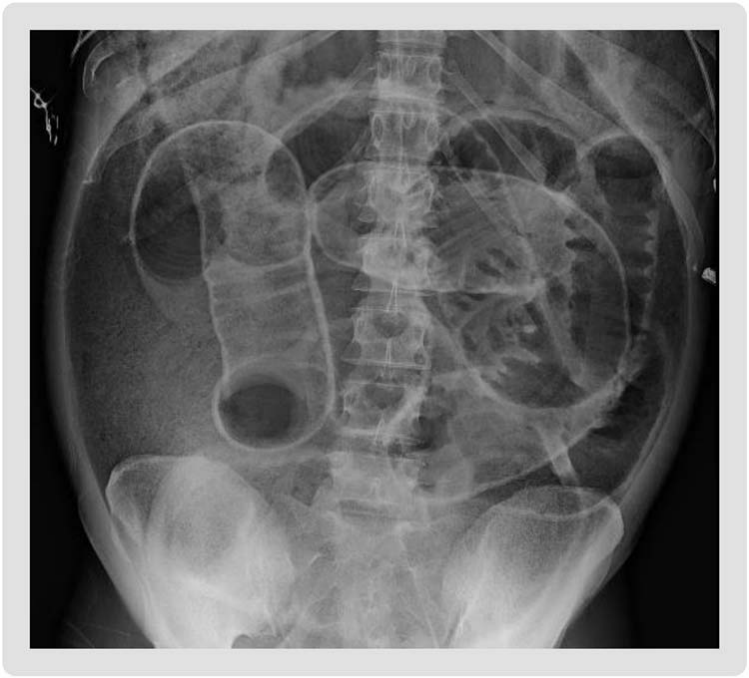
Supine abdominal plain film on admission demonstrating Rigler’s sign, bowel loops outlined by air, increasing suspicion for large volume pneumoperitoneum.

The patient is a 36-year-old female with a history of chronic constipation and toxic megacolon who presented in septic shock with severe abdominal distention and peritonitis. During the patient’s prior hospitalization, she presented with nausea, vomiting and abdominal distention. Computed tomography (CT) scan was performed and revealed stool in the colon without any obvious obstruction. She was diagnosed with chronic constipation with retained stool and thus underwent multiple fecal disimpactions during that hospitalization. The patient was seen by a gastroenterologist during the admission, who recommended colonoscopy as an outpatient. She would require further diagnostic work-up including Sitz marker study to diagnose possible colonic inertia [5]. The patient eventually had restoration of bowel function with laxatives after hospitalization Day 4 and was subsequently discharged. She continued to take her laxative for bowel movements, however, experienced no bowel movement for 7 days and had progressively worsening distension. She then presented to the hospital after developing sudden abdominal pain which prompted her to seek medical evaluation. On presentation, the patient was tachypneic, tachycardic and hypotensive. A stat abdominal X-ray demonstrated pneumoperitoneum, increasing urgency for the patient’s definitive surgical management (Fig. 1).

**Figure 2:**
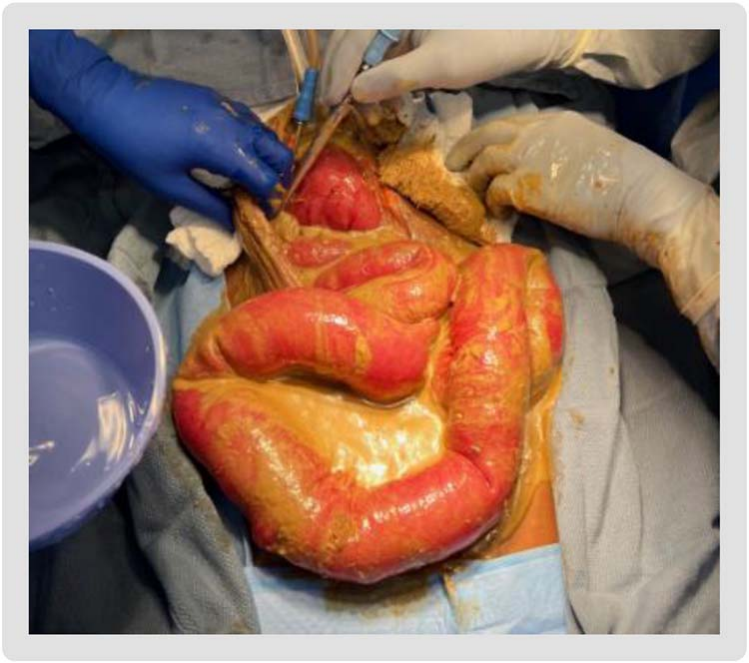
Diffuse feculent peritoneal contamination seen upon entering the abdomen.

**Figure 3:**
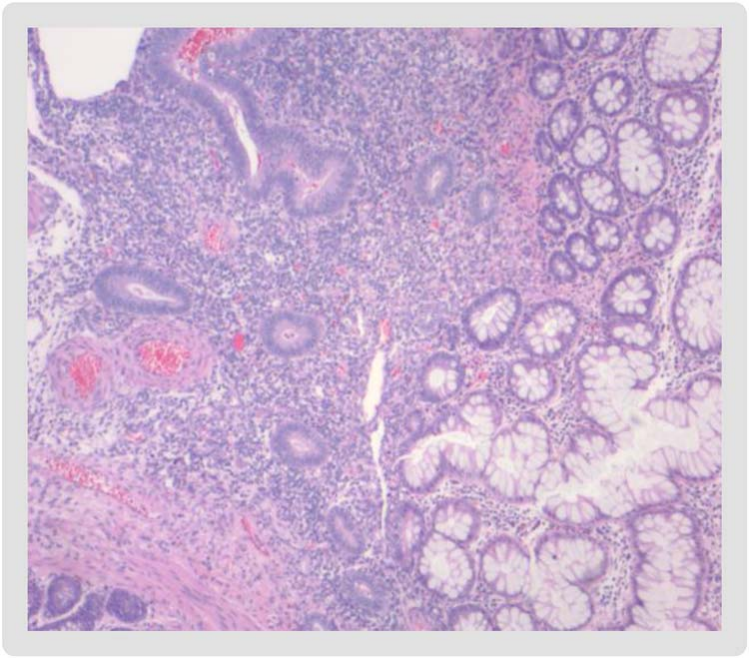
Endometrial mucosa (left) with intestinal glands from the large bowel (right).

The patient was emergently taken to the operating room with ongoing resuscitation efforts. Intraoperatively, she had diffused feculent peritoneal contamination (Fig. 2). An intraluminal mass in the distal sigmoid was appreciated; however, no free perforation was seen in the area. The remainder of the large intestine was examined and a perforation of the transverse colon was identified. The appendix was dilated to about 1.5 cm in diameter and appeared to be inflamed and adhered to the cecum with no signs of perforation. As a result of these intraoperative findings, the patient underwent a subtotal colectomy with abdominal washout, and was left in discontinuity. She was transferred to the intensive care unit (ICU) remaining intubated and sedated, on vasopressor support for hemodynamic instability. She was taken back to the operating room for a second-look exploratory laparotomy with washout on postoperative day 2 from the index procedure and again left in discontinuity. The patient was started on total parenteral nutrition (TPN) the following evening after this procedure for a likely prolonged recovery course. A third-look laparotomy, abdominal washout, end ileostomy creation and abdominal closure were performed on postoperative day 5 from the index procedure. The patient’s vital stability improved relatively quickly in the postoperative period and they were extubated the following day. The diet was slowly advanced and TPN discontinued due to return of bowel function through the ostomy 6 days after restoration of bowel continuity. The patient was on broad spectrum antibiotics to cover for gastrointestinal flora during the entire course of hospitalization. Pathological examination of the intraoperative specimen of the colon revealed endometriosis with transmural involvement and ulceration (Fig. 3). Additionally, the appendiceal sample also contained ectopic endometrial tissue.

## DISCUSSION

Endometriosis is the presence of viable endometrial gland tissues outside the uterine cavity. The most common sites are the pelvic peritoneum and pelvic organs. Extra-pelvic endometriosis is an uncommon presentation and sites include the bowel, appendix, lung, pleura and abdominal wall. Although the exact incidence of bowel endometriosis has not been adequately studied, it is estimated that between 3 and 37% of all endometriosis cases have some involvement of bowel [3]. Additionally, a vast majority of those cases involve the sigmoid colon (>90%) [4], as was seen in this patient. Endometriosis is characterized by cyclical pain that is exacerbated during menstruation. By comparison, extra-pelvic disease usually causes constant pain and varies in presentation based upon location of ectopic implantation. Ultrasound and CT are not diagnostic; however, ultrasound features of mixed echogenicity and lack of blood flow can be suggestive, whereas CT helps define anatomy and proximity to vessels. In this case, the patient had CT on a previous hospital admission and was of little utility, only showing signs of constipation. Treatment options include expectant management, hormonal therapy or complete surgical excision with avoidance of spillage to prevent recurrence. In this case, the patient had a complication of hollow viscus perforation and was managed based on this. Endometriosis should be included in the differential diagnosis of females who are of child-bearing age with chronic constipation and frequent partial small bowel obstruction symptoms.

## CONCLUSION

Symptoms of intestinal endometriosis mimic various intestinal diseases, thus it is difficult to diagnose preoperatively. Intestinal endometriosis should be considered when women of reproductive age have ambiguous symptoms and signs with preoperative evaluations. This case provides an excellent learning opportunity for a rare complication of a common disease that was difficult to diagnose but able to be treated under the usual standard of care.

## Data Availability

Case data is from patient’s chart at aforementioned hospital.
